# Investigating the transmission of baloxavir‐resistant influenza viruses from treated index patients to untreated household contacts in the BLOCKSTONE study

**DOI:** 10.1111/irv.13079

**Published:** 2023-01-18

**Authors:** Joanne Harding, Corrado Bernasconi, Sarah Williams, Steffen Wildum, Masahiro Kinoshita, Takeki Uehara, Aeron C. Hurt

**Affiliations:** ^1^ Roche Products Ltd. Welwyn Garden City UK; ^2^ F. Hoffmann‐La Roche Ltd. Basel Switzerland; ^3^ Shionogi & Co., Ltd. Osaka Japan

**Keywords:** antiviral agents, baloxavir, drug resistance, influenza, transmission, viral

## Abstract

In a post‐hoc analysis of the phase 3 BLOCKSTONE study (JapicCTI‐184180), we investigated household transmission of baloxavir‐resistant (PA/I38X) influenza viruses. Using baloxavir resistance rates from prior clinical trials and the rate of influenza transmission observed in the study, the predicted number of PA/I38X transmission events was 4.8, assuming wild type and PA/I38X viruses were equally transmissible. However, no PA/I38X viruses were observed. These results suggest a low potential for baloxavir‐resistant influenza virus transmission from treated to untreated individuals, potentially due to reduced viral/transmission fitness for PA/I38X viruses and/or low viral titres at the time when resistant viruses arise.

The influenza virus is subject to constant evolutionary change due to its high replication rate and lack of proofreading activity. This change can lead to the emergence of mutations that make the virus less susceptible to influenza antiviral drugs, resulting in antiviral resistance.^1^ In immunocompetent patients, influenza antiviral‐resistant viruses are typically transient and do not cause lasting infection, and they do not impact future treatment with the same antiviral.^1^ However, there is concern around the potential for widespread or sustained transmission of resistant viruses, which could result in an antiviral becoming no longer suitable for treatment of influenza cases in the community.^1^ Baloxavir marboxil (baloxavir) is a first‐in‐class influenza antiviral that inhibits cap‐dependent endonuclease activity of the influenza virus^2^ and has been approved as both a single‐dose treatment and post‐exposure prophylaxis for influenza in over 70 countries worldwide.^3^

Resistance to baloxavir is most frequently caused by substitutions at isoleucine 38 in the viral polymerase acidic subunit of the influenza RNA polymerase (PA/I38X).^1^ Treatment‐emergent baloxavir resistant viruses have been observed in phase 3 pivotal clinical trials, with rates of 5% to 10% in adults and 19% in children.^4^, ^5^, ^6^ To date, sustained transmission of baloxavir‐resistant viruses has not been observed in any population, although there have been reports of sporadic transmission of baloxavir‐resistant viruses in Japan.^7^ There is a need for a greater understanding of the potential for transmission of baloxavir‐resistant viruses.

To examine the likelihood of baloxavir‐resistant virus transmission relative to wild type, we conducted a post‐hoc analysis of the BLOCKSTONE study (JapicCTI‐184180). We compared the number of transmission events of baloxavir‐resistant viruses in the study with those predicted to occur, using influenza household transmission rates from the study, baloxavir treatment‐emergent resistance rates from across baloxavir clinical trials and the assumption of equivalent viral fitness for all influenza viruses. In the BLOCKSTONE study, household contacts (HHCs) who lived with an index patient (IP) with influenza were randomized to receive either baloxavir or placebo as prophylaxis. As BLOCKSTONE was conducted in Japan where treating influenza with antivirals is standard of care, all IPs in the study were treated with antivirals including baloxavir.^8^ This provided the opportunity to investigate transmission of baloxavir‐resistant viruses from baloxavir‐treated IPs to HHCs who received placebo prophylaxis.

IPs were included if they had been treated with baloxavir, had a polymerase chain reaction (PCR)‐confirmed influenza infection and lived with at least one HHC. HHCs must have received placebo, been PCR‐negative at baseline and if they developed influenza, had a virus that matched the type and subtype of that of their IP (suggesting the virus was transmitted within that household). Per the BLOCKSTONE protocol, post‐baseline nasopharyngeal swab samples were obtained from HHCs at day 5, day 11 and days 1 to 10 (if influenza was suspected), and Sanger sequencing was carried out on the first PCR‐positive sample to identify PA/I38X substitutions. This strategy was expected to detect all PA/I38X transmission cases. Overall, 152 baloxavir‐treated IPs and 172 HHCs were included in the analysis. IPs were predominantly aged <12 years and had A/H3N2 or A/H1N1 infections (Table 1).

**TABLE 1:** Breakdown of baloxavir‐treated index patients with household contacts receiving placebo in the BLOCKSTONE study by age and viral subtype of influenza infection

	A/H3N2	A/H1N1	B	Mixed infection^a^
Overall (*n* = 152)	77	71	2	2
<12 years (*n* = 90)	40	47	2	1
≥12 years (*n* = 62)	37	24	0	1

^a^ A/H1N1 and A/H3N2.

No PA/I38X viruses were identified in any IPs at day 1. As samples were not collected from IPs after day 1, it was not possible to know whether treatment‐emergent PA/I38X viruses developed in these patients after this timepoint. Therefore, the incidence of treatment‐emergent PA/I38X in IPs was predicted based on estimates from pooled baloxavir clinical trial data stratified by age group (<12 or ≥12 years of age) and virus type/subtype (A/H1N1, A/H3N2 and B) (Table 2). This incidence was multiplied by the number of eligible IPs in each stratum to give a predicted number of IPs with PA/I38X viruses of 19.7 (Table 3).

**TABLE 2:** Rates of PA/I38X by age and influenza subtype from pooled baloxavir studies

Pooled ITTI population^a^	A/H3N2	A/H1N1	B
<12 years	25.9% (45/174)	12.2% (6/49)	0% (0/41)
≥12 years	8.0% (49/611)	2.1% (5/235)	0.7% (2/287)

Abbreviations: ITTI, intention‐to‐treat infected; PA, polymerase acidic.

^a^ Pooled data from the following phase 3 studies: CP40559 (miniSTONE‐1, NCT03653364; interim data); CP40563 (miniSTONE‐2, NCT03629184); T0822 (Japanese paediatric tablet study, JapicCTI‐163417); T0831 (CAPSTONE‐1, NCT02954354); T0832 (CAPSTONE‐2, NCT02949011); T0833 (Japanese paediatric granule study, JapicCTI‐173811); T0835 (Japanese paediatric double dose study, JapicCTI‐194577); and one phase 2 study T0821 (otherwise healthy, JapicCTI‐153090). Patients with mixed infection (A/H1N1 and A/H3N2) were counted once in the total number of patients and once by each virus type/subtype category.

**TABLE 3:** Predicted number of index patients infected with PA/I38X viruses in the BLOCKSTONE study

	A/H3N2	A/H1N1	B	Mixed infection^a^	Total
Overall (*n* = 152)	13.4	6.2	0	0.1	19.7
<12 years (*n* = 90)	10.4	5.7	0	0.1	16.2
≥12 years (*n* = 62)	3.0	0.5	0	0	3.5

Abbreviation: PA, polymerase acidic.

^a^ A/H1N1 and A/H3N2.

To calculate the number of expected PA/I38X virus infections in HHCs, the transmission rate of wild‐type viruses was first determined. Among those included in the analysis, IPs were associated with a single infected HHC in most cases; therefore, the analysis focused on household‐level transmission. The rate of household transmission events was calculated by dividing the number of households with infected HHCs (39 HHCs from 37 households) by the number of baloxavir‐treated IPs (*n* = 152). This resulted in a household transmission rate of 24.3%.

For the purposes of this analysis, we assumed that there was equal transmissibility of viruses with treatment‐emergent PA/I38X substitutions and wild‐type viruses and that the IPs had a level of treatment‐emergent PA/I38X virus similar to pooled PA/I38X rates from baloxavir clinical trials. Therefore, the predicted number of influenza‐infected households with PA/I38X virus transmission was 4.8, which was determined by multiplying the expected number of IPs with PA/I38X viruses (19.7) by the rate of household transmission events (24.3%). However, based on actual sequence analysis, no PA/I38X variants were identified among HHCs, apart from in two individuals from a single household, but only after they received rescue treatment with baloxavir. These two patients were influenza negative at baseline and subsequently tested positive for wild‐type virus on day 3, before testing positive for PA/I38X virus on day 5 after rescue treatment with baloxavir. They were therefore included in the analysis as wild‐type transmission events, rather than PA/I38X transmission events. The probability of observing no PA/I38X transmission events in our household sample when 4.8 cases were predicted was 0.0077, based on a model for independent transmission events (Bernoulli trials) (Table 4). This probability would remain <0.05 when the predicted number of household transmission events was reduced to 3 (63% of the expected value). Alternative hypothetical scenarios are shown in Table 4, exploring the effect of higher or lower rates of PA/I38X in IPs; these alternative scenarios showed that it was only if very low rates of PA/I38X viruses were observed in IPs that zero cases in HHCs could be reasonably expected. Taken together, these probabilities suggested that the assumption of equivalent transmission fitness of PA/I38X viruses and wild‐type viruses was likely incorrect and/or that resistant viruses were occurring late in the course of infection when viral titres were low. Overall, these findings strongly suggest that the likelihood of transmission of PA/I38X viruses is lower than for wild‐type viruses.

**TABLE 4:** Probability of detecting no baloxavir‐resistant viruses in households, assuming different rates of PA/I38X viruses in index patients

Assumed PA/I38X rate in IPs	Predicted number of IPs with PA/I38X viruses	Expected number of households with PA/I38X viruses	Probability of detecting no PA/I38X viruses in households^a^
13%	19.7	4.8	0.0077
20%^b^	30.4	7.4	0.0005
5%^b^	7.6	1.9	0.1555

Abbreviations: IP, index patient; PA, polymerase acidic.

^a^ Based on a binomial model.

^b^ Alternative assumptions for the rate of PA/I38X in IPs.

Regardless of assumptions about PA/I38X viruses in IPs, the fact that no PA/I38X transmission was observed in our sample of 152 households allowed the overall risk of this event to be calculated: Based on a binomial distribution, there was 95% confidence that the probability of IPs transmitting PA/I38X viruses to their HHCs was <0.02.

These results suggest a low potential for baloxavir‐resistant virus transmission from baloxavir‐treated individuals with influenza to untreated individuals. A possible explanation for the low rate of transmission of baloxavir‐resistant viruses observed in the study is that PA/I38X‐substituted viruses may have reduced intrinsic fitness relative to wild type that decreases their ability to transmit. This is supported in part by a recent study of baloxavir‐resistant influenza virus fitness in ferrets, where PA/I38T substitutions reduced fitness of A/H1N1 and A/H3N2 viruses in competition with wild‐type viruses, although it should be noted that the reduction in fitness was modest.^9^ Whilst ferret studies provide some insights into the fitness and transmissibility of influenza viruses, the translatability of the animal data to a human household/population is limited; therefore, learnings from human clinical studies such as this remain highly relevant.

A second possible explanation for the lack of transmission of baloxavir‐resistant viruses is that these viruses arise later in the course of infection in treated IPs, when viral titres are lower than peak viral loads.^1^ Therefore, the chances that these viruses are transmitted to others may be lower than if they arose when viral loads were high. Surveillance studies continue to report very low or no incidence of baloxavir‐resistant viruses being detected in untreated patients to date.^10^, ^11^, ^12^, ^13^, ^14^

One limitation of this analysis is that the BLOCKSTONE study design did not allow for direct assessment of transmission of baloxavir‐resistant viruses from treated IPs, as no virology samples were collected from IPs after day 1 of the study. This will be investigated in the ongoing CENTERSTONE trial (NCT03969212), which is evaluating the efficacy of baloxavir in reducing transmission of influenza from treated IPs to untreated HHCs.^15^ Another potential limitation is that it was assumed that all transmission events occurred from the IP to the HHC (or from within the IP's household) and that no viruses were transmitted from outside the household.

In conclusion, the results of this post‐hoc analysis suggest that baloxavir‐resistant PA/I38X viruses may transmit less frequently than wild‐type influenza viruses; however, further validation is needed. The ongoing CENTERSTONE trial will provide additional insights into transmission of baloxavir‐resistant viruses.

## CONFLICTS OF INTEREST

The authors declare the following financial interests/personal relationships, which may be considered as potential competing interests: Joanne Harding and Sarah Williams are employees and shareholders of Roche Products Ltd. Aeron C. Hurt and Steffen Wildum are employees and shareholders of F. Hoffmann‐La Roche Ltd. Corrado Bernasconi is a contractor for F. Hoffmann‐La Roche Ltd. Masahiro Kinoshita and Takeki Uehara are employees of Shionogi & Co., Ltd.

## ETHICS APPROVAL AND PATIENT CONSENT

The BLOCKSTONE study was conducted according to the principles of the Declaration of Helsinki and the International Council for Harmonisation guidelines for Good Clinical Practice. The study protocol was approved by an institutional review board at each trial site, and all index patients and household contacts (or their legal representatives) provided written informed consent.

## AUTHOR CONTRIBUTIONS

**Joanne Harding:** Conceptualization; writing‐original draft; writing‐review and editing. **Corrado Bernasconi:** Formal analysis; writing‐original draft; writing‐review and editing. **Sarah Williams:** Conceptualization; formal analysis; writing‐original draft; writing‐review and editing. **Steffen Wildum:** Conceptualization; writing‐original draft; writing‐review and editing. **Masahiro Kinoshita:** Conceptualization; writing‐original draft; writing‐review and editing. **Takeki Uehara:** Conceptualization; writing‐original draft; writing‐review and editing. **Aeron C. Hurt:** Conceptualization; writing‐original draft; writing‐review and editing.

### PEER REVIEW

The peer review history for this article is available at https://publons.com/publon/10.1111/irv.13079.

## Data Availability

The data that support the findings of this study are available from the corresponding author upon reasonable request.

## References

[1] Holmes EC , Hurt AC , Dobbie Z , Clinch B , Oxford JS , Piedra PA . Understanding the impact of resistance to influenza antivirals. Clin Microbiol Rev. 2021;34(2). doi:10.1128/CMR.00224-20 PMC7950363 33568554

[2] Noshi T , Kitano M , Taniguchi K , et al. In vitro characterization of baloxavir acid, a first‐in‐class cap‐dependent endonuclease inhibitor of the influenza virus polymerase PA subunit. Antiviral Res. 2018;160:109‐117. doi:10.1016/j.antiviral.2018.10.008 30316915

[3] Roche . Roche announces U.S. FDA approval of Xofluza to treat influenza in children aged five years and older. 2022. Accessed October 18, 2022. https://www.roche.com/media/releases/med-cor-2022-08-12

[4] Baker J , Block SL , Matharu B , et al. Baloxavir marboxil single‐dose treatment in influenza‐infected children: a randomized, double‐blind, active controlled phase 3 safety and efficacy trial (miniSTONE‐2). Pediatr Infect Dis J. 2020;39(8):700‐705. doi:10.1097/INF.0000000000002747 32516282 PMC7360097

[5] Hayden FG , Sugaya N , Hirotsu N , et al. Baloxavir marboxil for uncomplicated influenza in adults and adolescents. N Engl J Med. 2018;379(10):913‐923. doi:10.1056/NEJMoa1716197 30184455

[6] Ison MG , Portsmouth S , Yoshida Y , et al. Early treatment with baloxavir marboxil in high‐risk adolescent and adult outpatients with uncomplicated influenza (CAPSTONE‐2): a randomised, placebo‐controlled, phase 3 trial. Lancet Infect Dis. 2020;20(10):1204‐1214. doi:10.1016/S1473-3099(20)30004-9 32526195

[7] NIID . Detection of antiviral drug‐resistant viruses in Japan during the 2018/2019 influenza season. 2019. Accessed October 18, 2022. https://www.niid.go.jp/niid/images/flu/resistance/20191128/dr18-19e20191126-1.pdf

[8] Ikematsu H , Hayden FG , Kawaguchi K , et al. Baloxavir marboxil for prophylaxis against influenza in household contacts. N Engl J Med. 2020;383:309‐320. doi:10.1056/NEJMoa1915341 Protocol available at: https://www.nejm.org/doi/suppl/10.1056/NEJMoa1915341/suppl_file/nejmoa1915341_protocol.pdf 32640124

[9] Lee LY , Zhou J , Koszalka P , et al. Evaluating the fitness of PA/I38T‐substituted influenza A viruses with reduced baloxavir susceptibility in a competitive mixtures ferret model. PLoS Pathog. 2021;17(5):e1009527. doi:10.1371/journal.ppat.1009527 33956888 PMC8130947

[10] WHO . Recommended composition of influenza virus vaccines for use in the 2020 southern hemisphere influenza season. 2019. Accessed October 18, 2022. https://cdn.who.int/media/docs/default-source/influenza/who-influenza-recommendations/vcm-southern-hemisphere-recommendation-2020/201909-recommendation.pdf?sfvrsn=f34905d9_13&download=true

[11] WHO . Recommended composition of influenza virus vaccines for use in the 2020–2021 northern hemisphere influenza season. 2020. Accessed October 18, 2022. https://cdn.who.int/media/docs/default-source/influenza/who-influenza-recommendations/vcm-northern-hemisphere-recommendation-2020-2021/202002-recommendation.pdf?sfvrsn=6868e7b3_21&download=true

[12] WHO . Recommended composition of influenza virus vaccines for use in the 2021 southern hemisphere influenza season. 2020. Accessed October 18, 2022. https://cdn.who.int/media/docs/default-source/emergency-preparedness/global-influenza-programme/recommended-composition-of-influenza-virus-vaccines/202009-recommendation.pdf?sfvrsn=1331fa94_2&download=true

[13] WHO . Recommended composition of influenza virus vaccines for use in the 2021–2022 northern hemisphere influenza season. 2021. Accessed October 18, 2022. https://cdn.who.int/media/docs/default-source/influenza/who-influenza-recommendations/vcm-northern-hemisphere-recommendation-2021-2022/202102_recommendation.pdf?sfvrsn=2af603d8_12&download=true

[14] WHO . Recommended composition of influenza virus vaccines for use in the 2022–2023 northern hemisphere influenza season. 2022. Accessed October 18, 2022. https://cdn.who.int/media/docs/default-source/influenza/who-influenza-recommendations/vcm-northern-hemisphere-recommendation-2022-2023/202202_recommendation.pdf?sfvrsn=5c88e006_13&download=true

[15] ClinicalTrials.Gov. Study to assess the efficacy of baloxavir marboxil versus placebo to reduce onward transmission of influenza A or B in households. 2022. Accessed October 18, 2022. https://clinicaltrials.gov/ct2/show/NCT03969212

